# Are tongue flaps effective in the closure of palatal fistulas? A systematic review and meta-analysis

**DOI:** 10.1007/s10006-025-01392-w

**Published:** 2025-05-19

**Authors:** Feras AlMofreh AlQahtani, Sam Kuriadom, Michalis Mastrogeorgiou, Abubaker Abualgasim, Hanan MR Shokier, Shadia A. ElSayed

**Affiliations:** 1 Department of Oral Surgery, Queen Mary University of London, London, UK; 2 College of Dentistry, Center of Medical and Bio-allied Health Sciences Research, Ajman University, Ajman, United Arab Emirates; 3 Department of Ears, Nose, Throat & Oral and Maxillofacial Surgery, Theageneio Cancer Hospital, Thessaloniki, Greece; 4 Department of Oral and Maxillofacial Surgery, AlMana General Hospital Group, Dammam, Kingdom of Saudi Arabia; 5 Department of Oral and Maxillofacial Surgery and Diagnostic Science, Faculty of Dentistry, Umm Al- Qura University, Makkah, Kingdom of Saudi Arabia; 6 Department of Oral and Maxillofacial Diagnostic Sciences, College of Dentistry, Health and Life Research Center, Taibah University, Madinah, Kingdom of Saudi Arabia

**Keywords:** Tongue flap, Palatal fistula, Closure, Systematic review, Meta-analysis

## Abstract

**Purpose:**

Is to Sytematically review the available evidence on the effectiveness of tongue flaps in the Closure of Palatal Fisutlas.

**Methods:**

The study protocol was developed in accordance with the Preferred Reporting Items for Systematic Reviews and Meta-Analysis Protocols, and it was registered with the International Prospective Register of Systematic Reviews under registration number CRD42023397034.

**Results:**

Our search strategy yielded 587 articles. Of these, 150 were duplicate studies, and 437 were screened. Of these studies, 392 studies were excluded based on their titles and abstracts. 45 records were assessed for eligibility in which 29 were excluded as they did not meet the inclusion criteria. Finally, 16 studies met the criteria for inclusion, and they were critically reviewed.

**Conclusion:**

This study highlights that tongue flaps, particularly dorsal tongue flaps (DTF) and posterior tongue flaps (PTF), are effective options for palatal fistula closure, demonstrating high success rates and a favorable complication profile. Nonetheless, additional research is necessary to explore the potential of these techniques for closing oroantral fistulas (OAF). Further investigations should employ randomized controlled trials with larger patient cohorts and extended follow-up periods to comprehensively evaluate the efficacy, complication rates, and long-term outcomes of DTF and PTF in OAF treatment.

## Introduction

Palatal fistulas arise from surgical operations performed in the palate area due to various reasons among which include trauma, infection and iatrogenic which manifests with intense signs and symptoms of food and/or air coming through the nasal, maxillary, or oral cavity and rebound sinus infection [1, 2]. Chief complaint is the over excess regression of fluids and food in the nasal cavity, leading to dysphagia, nasal speech and impaired articulation [3]. Surgical and non-surgical approaches can be implemented for the management of the anterior palatal fistula, likewise intraoral obturators and local either distant site flaps, such as arm, thorax, head and neck, nasal septum, cheek region [4]. The regional flaps exhibit complications, such as scarring, tension upon closure leading to wound dehiscence and tissue necrosis, while the distant ones require longer periods of hospital stay and bring along the intra- and post-operative difficulties of flap transportation as well as fixation [2, 5]. Thus, the repairment of the oro-nasal communication and the formed palatal fistula, is of great difficulty and requires extensive anatomy comprehension and detailed planning. As far as the designated flap is concerned, there are a few available in our arsenal, among which temporal muscle flap, mucoperiosteal and pedicled tongue flap [2, 5, 6]. The latter one, the pedicled tongue flap, is the flap of choice especially in the setting of fistulae underlying in clefts bigger than 1 cm due to its plethoric characteristics that of mobility, anatomical site proximity, adequate width, satisfying neovascularization and simple technique with lesser complications regarding the anatomic donor site [7]. It was first described in 1091 by Eiselberg and was put into detail by Guerrero-Santos and Altamirano in 1966 [8]. Under the umbrella of this flap, two possible techniques can be utilized depending on the targeted anatomical site of the fistula, with one being the anterior flap, with satisfying mobilization and elasticity, for the hard palate and anterior oral mucosa and the posterior one, which is the overall treatment of choice due to its vascular properties, for the soft palate, retromolar area, and posterior oral mucosa [7, 9]. The confluent vascularity allocated in the submucous layers of the tongue allows for greater predictability and stability of outcomes utilizing every single pattern in any direction with quite the versatility [10]. The surgical closure of the defect will lead to prevention of faulty odor, appearance of chronic sinus and nasal infection and improvement of masticatory and speech function as well [11]. Postoperatively, the pedicled tongue flap exhibits a series of challenges and misshapen such as flap separation and the difficulty of its immobilization to the site due to the increased muscular layering of the tongue and its subsequent mobility during mastication, speech, and other everyday functions. These challenges, bring forward the option of fixation techniques to overcome them, among which K-wire technique, intermaxillary, ‘’parachuting and anchoring ‘’ technique and flap ‘’take’’ method [12–15]. The aim of this study is to sytematically review the available evidence on the effectiveness of tongue flaps in the reconstruction of palatal fistulas and to appraise the usage of the tongue flap in the management of palatal fistula and contemplate upon its favorable prognosis and outcome in concern to the challenges that it bears within, setting the technique itself under scrutiny for better clinical impact.

## Materials and methods

### Protocol and registration

The protocol was developed per the Preferred Reporting Items for Systematic Reviews and Meta-Analysis Protocols [16] (PRISMA-P) and registered with the International Prospective Register of Systematic Reviews (PROSPERO) under the registration number CRD42023397034.

### Information sources and search strategy

The electronic databases of PubMed, Web of Science and Scopus were systematically reviewed to identify relevant studies. All articles published in English from first registry up until July 2024 were reviewed. The terms selected for the purposes of this search were “Dorsal Tongue Flap” “Tongue Flaps” and” Palatal Fistula” with the use of the boolean operator AND in the different registries (Table 1). We then used the PICO framework: **P** (Patient Population), **I** (Intervention or Exposure—in the case of observational studies), **C** (Comparison) and **O** (Outcomes). In this systematic review, the PICO approach involved Population (Patients with palatal fistulas), Exposure (Tongue flap), Comparison (Palatal fistulas before and after tongue flap) and Outcome (If Tongue flaps are effective in the closure of palatal fistulas).

**Table 1:** Search strategy

Databases	Keyword combinations	Total results
PUBMED	(((((((tongue flap[Text Word]) OR (tongue flap[Title/Abstract])) OR (tongue flap)) AND (fistula, palatal[MeSH Terms])) OR (palatal fistula[Text Word])) OR (palatal fistula[Title/Abstract])) OR (tongue flap[Text Word])) AND (cleft palate[Text Word])	223
Scopus	( KEY ( tongue AND flap ) OR TITLE-ABS-KEY ( tongue AND flap ) OR ALL ( tongue AND flap ) AND KEY ( fistula, AND palatal ) OR TITLE-ABS-KEY ( palatal AND fistula ) OR ALL ( palatal AND fistula ) OR KEY ( tongue AND flap ) AND TITLE-ABS-KEY ( cleft AND palate ) ) AND ( LIMIT-TO ( LANGUAGE, “English” ) )	267
Web of Science	(((((((ALL=(tongue flap)) OR KP=(tongue flap)) OR AK=(tongue flap)) AND ALL=(fistula, palatal)) OR KP=(palatal fistula)) AND ALL=(palatal fistula)) OR ALL=(tongue flap)) AND ALL=(cleft palate)	97

### Data collection

Data screening and extraction was performed by two authors (F.M.Q) and (M.M) using the standardised data extraction form Cohen’s kappa (κ) index was calculated to evaluate the level of agreement between the two reviewers. Disagreements were resolved through a third reviewer (SK). Full-text articles were retrieved and analyzed by three reviewers. Data collected from each study included: Author, year of publication, design of the included study, number of patients, size of palatal fistula, location of Palatal fistula, follow up interval and complications (Table 2).

**Table 2:** Summary of study characteristics

Author/ Year	Study Type	Number of Patients	Site of Donor Flap	Size of Palatal Fistula	Location of Palatal Fistula	Follow up	Success	Complications
N. Gupta/ 2019	Prospective	20	Dorsal Anterior Tongue	5 × 10 - 15 × 20 mm	Anterior Palate	1 Year	18 Flaps	Flap Detachment (1) Flap Necrosis (1)
F. Kocaaslan/ 2020	Retrospective	34	Dorsal Anterior Tongue	> 1 cm	Anterior Palate	4 Weeks	25 Flaps	Flap Detachment (9)
Candemir Ceran/ 2012	Retrospective	6	Dorsal Anterior Tongue	*	Anterior Palate	6 Months	6 Flaps	Temporary Anterior Venous Congestion (2)
N. Busic/1989	Retrospective	19	Dorsal Anterior Tongue	> 1.5 cm	Anterior Palate (17) Posterior Palate (2)	3 Weeks	17 Flaps	Total Flap Necrosis (1) Partial Marginal Necrosis of the Flap (1)
A.Habib/2015	Prospective	30	Dorsal Anterior Tongue	1.58 cm	Anterior Palate	4 Months − 24 Months	30 Flaps	No Complications
SPS Sudhi/2013	Prospective	20	Dorsal Anterior Tongue	> 5 mm	Anterior Palate	3 Months– 6 Months	18 Flaps	Decrease in Hyper nasality (16) Necrosis of the Tongue Flap (2) Regurgitation of Fluid/Food (2) Revenant Fistula (2)
Amin Rahpeyma /2015	Retrospective	7	Posterior Lateral Tongue Flap	*	Anterior Palate	2–7 Years	6 Flaps	Asymmetrical Tongue Shape (6)
Antonio Guedes / 1992	Retrospective	12	Dorsal Anterior Tongue	*	Anterior Palate	6–15 Months	12 Flaps	Bleeding from the flap (2) Recurrent Fistula (1)
S. Abdollahi / 2008	Retrospective	23	Dorsal Anterior Tongue	≥ 2 cm	Anterior Palate	28 ± 4 months	20 Flaps	Partial Rejection (3) Complete Rejection (3)
Dionelys Barazarte / 2020	Retrospective	20	Dorsal Anterior Tongue	8 × 12 cm to 10 × 15 cm	Unilateral Complete Cleft Lip and Palate, Bilateral Complete Cleft Lip and Palate	4–24 months	20 Flaps	Partial dehiscence (8)
Abdulla K. Alsalman / 2016	Retrospective	5	Dorsal Anterior Tongue	1.5 × 1.5 cm to 4.5 × 2 cm	Hard Palate, Junction of the Primary and Secondary Palate	18 months	5 Flaps	None
Ravi Kumar Mahajan / 2018	Retrospective	153	Not specified	< 2 mm to > 5 mm	Anterior Palate	6 months	77 Flaps	Flap dehiscence (3)
Advait Prakash / 2018	Retrospective	18	Anterior Dorsal Tongue	2 cm × 1.5 cm to 5.0 cm × 3 cm	Anterior Palate	6 months	18 Flaps	None
Julian D. Meneses Argalle / 2023	Cohort	30	Anterior Dorsal Tongue	15–35 mm	Hard Palate, Junction of Primary and Secondary Palate, Lingual Alveolar	12 months	29 Flaps	Partial Necrosis (1)
Carlos Giugliano / 2022	Retrospective	24	Anterior Dorsal Tongue	2.5 cm	Anterior Palate	4 years	21 Flaps	Dehiscence (2), Persistent residual fistula (1)
Sathish M.S. Vasishta / 2012	Retrospective	40	Anterior Dorsal Tongue	11.57 × 13.58 mm	Primary and Secondary Palate, Hard Palate, Junction of the Soft and Hard Palate	15 months	37 Flaps	Bleeding (1), Dehiscence (2), Sloughing (1), Detachment (2), Recurrence of fistula (3)

### Eligibility criteria

#### Inclusion criteria

Original Articles including Randomised Clinical Trials, Prospective, Retrospective, Cohort studies. Articles with full-text availability rather than just an abstract. All articles reporting patients with a palatal fistula treated by tongue flaps were included.

#### Exclusion criteria

Non-English articles, Systematic or Literature Reviews articles, Case reports and case series studies were excluded. Any article reporting patients with a palatal fistula treated by any mean other that tongue flaps was excluded. Studies that failed to report a follow up period were excluded.

### Risk of bias quality assessment

For the original non-randomized studies quality was assessed using the Newcastle–Ottawa quality assessment scale [17] (Table 3).

**Table 3:** Newcastle Ottawa scale risk of Bias (RoB) assessment for Non-Randomized studies

Study ID	Selection			Comparability *	Outcome		Total
	Representatives of exposed Cohort (*)	Selection of non- exposed cohort (*)	Ascertainments of exposure (*)	(**)	Assessment of Outcome (*)	Adequacy of Follow Up (*)	Out of 7
N. Gupta/ 2019	*	*	-	**	*	*	6
F. Kocaaslan/ 2020	*	*	-	**	*	*	6
Candemir Ceran/ 2012	*	*	-	**	*	*	6
N. Busic/1989	*	*	-	**	*	*	6
A.Habib/2015	*	*	-	**	*	*	6
SPS Sudhi/2013	*	*	-	**	*	*	6
Amin Rahpeyma /2015	*	*	-	**	*	*	6
Antonio Guedes / 1992	*	*	-	**	*	*	6
S. Abdollahi / 2008	*	*	-	**	*	*	6
Dionelys Barazarte / 2020	*	*	-	**	*	*	6
Abdulla K. Alsalman / 2016	*	*	-	**	*	*	6
Ravi Kumar Mahajan / 2018	*	*	-	**	*	*	6
Advait Prakash / 2018	*	*	-	**	*	*	6
Julian D. Meneses Argalle / 2023	*	*	-	**	*	*	6
Carlos Giugliano / 2022	*	*	-	**	*	*	6
Sathish M.S. Vasishta / 2012	*	*	-	**	*	*	6

### Certainty of the level of evidence

The GRADE tool [18] (Grading of Recommendations, Assessment, Development, and Evaluation) was used to assess the quality of a body of evidence (GRADE working group). The quality of evidence was rated per outcome into one of four categories (high, moderate, low, and very low) (Table 4).

**Table 4:** Grades assessment of certainty level of evidence

Author / Year	Risk of Bias	Inconsistency	Indirectness	Imprecision	Publication Bias	Overall Level of Evidence
N. Gupta/ 2019	No Serious Limitation	No Serious Inconsistency	No Serious Indirectness	No Serious Imprecision	No Bias Detected	High
F. Kocaaslan / 2020	No Serious Limitation	No Serious Inconsistency	No Serious Indirectness	No Serious Imprecision	No Bias Detected	High
Candemir Ceran/ 2012	No Serious Limitation	No Serious Inconsistency	No Serious Indirectness	No Serious Imprecision	No Bias Detected	High
N. Busic/ 1989	No Serious Limitation	No Serious Inconsistency	No Serious Indirectness	No Serious Imprecision	No Bias Detected	High
A.Habib/ 2015	No Serious Limitation	No Serious Inconsistency	No Serious Indirectness	No Serious Imprecision	No Bias Detected	High
SPS Sudhi/ 2013	No Serious Limitation	No Serious Inconsistency	No Serious Indirectness	No Serious Imprecision	No Bias Detected	High
Amin Rahpeyma / 2015	No Serious Limitation	No Serious Inconsistency	No Serious Indirectness	No Serious Imprecision	No Bias Detected	High
Guzel / 2000	No Serious Limitation	No Serious Inconsistency	No Serious Indirectness	No Serious Imprecision	No Bias Detected	High
Antonio Guedes / 1992	No Serious Limitation	No Serious Inconsistency	No Serious Indirectness	No Serious Imprecision	No Bias Detected	High
S. Abdollahi / 2008	No Serious Limitation	No Serious Inconsistency	No Serious Indirectness	No Serious Imprecision	No Bias Detected	High
Dionelys Barazarte / 2020	No Serious Limitation	No Serious Inconsistency	No Serious Indirectness	No Serious Imprecision	No Bias Detected	High
Abdulla K. Alsalman / 2016	No Serious Limitation	No Serious Inconsistency	No Serious Indirectness	No Serious Imprecision	No Bias Detected	High
Ravi Kumar Mahajan / 2018	No Serious Limitation	No Serious Inconsistency	No Serious Indirectness	No Serious Imprecision	No Bias Detected	High
Advait Prakash / 2018	No Serious Limitation	No Serious Inconsistency	No Serious Indirectness	No Serious Imprecision	No Bias Detected	High
Julian D. Meneses Argalle / 2023	No Serious Limitation	No Serious Inconsistency	No Serious Indirectness	No Serious Imprecision	No Bias Detected	High
Carlos Giugliano / 2022	No Serious Limitation	No Serious Inconsistency	No Serious Indirectness	No Serious Imprecision	No Bias Detected	High
Sathish M.S. Vasishta / 2012	No Serious Limitation	No Serious Inconsistency	No Serious Indirectness	No Serious Imprecision	No Bias Detected	High

### Data analysis

The quantitative synthesis was performed using Metapreg package of Stata statistical software (Statacorp., College station, Texas, USA, version 17). The overall mean success rate of fistula closure using tongue flaps was estimated using a random effects model to account for the significant heterogeneity observed across the included studies. Fixed effects models were used to pool the results due to the low heterogeneity observed across the included studies. The heterogeneity across the included studies was assessed using Higgins test I^2^ [19]. Likelihood ratio test was performed to check whether the estimates for a particular outcome measure fits better in random effects or fixed effects model. Additionally, meta-regression analysis was performed to evaluate the impact of various predictors, such as study design, length of palatal fistula, width of the palatal fistula and follow-up period, on the overall pooled estimate for the success of palatal fistula closure with tongue flaps. Egger’s test [20] was performed to assess the presence of small-study effects across the included studies. The presence of publication bias was assessed using contour enhanced funnel plot with levels of significance set at 0.10, 0.05 and 0.01 levels.

The study also employed Bayesian meta-analysis methods using the BayesFactor package (version 0.9.12–4.2) in R statistical software (R computing, Vienna, Austria, version 4.3.3) to evaluate improvements in speech intelligibility, hypernasality, and nasal emission at one month, six months, and one-year post-intervention after palatal fistula closure with tongue flaps.

## Results

### Search of the literature

**Fig. 1:**
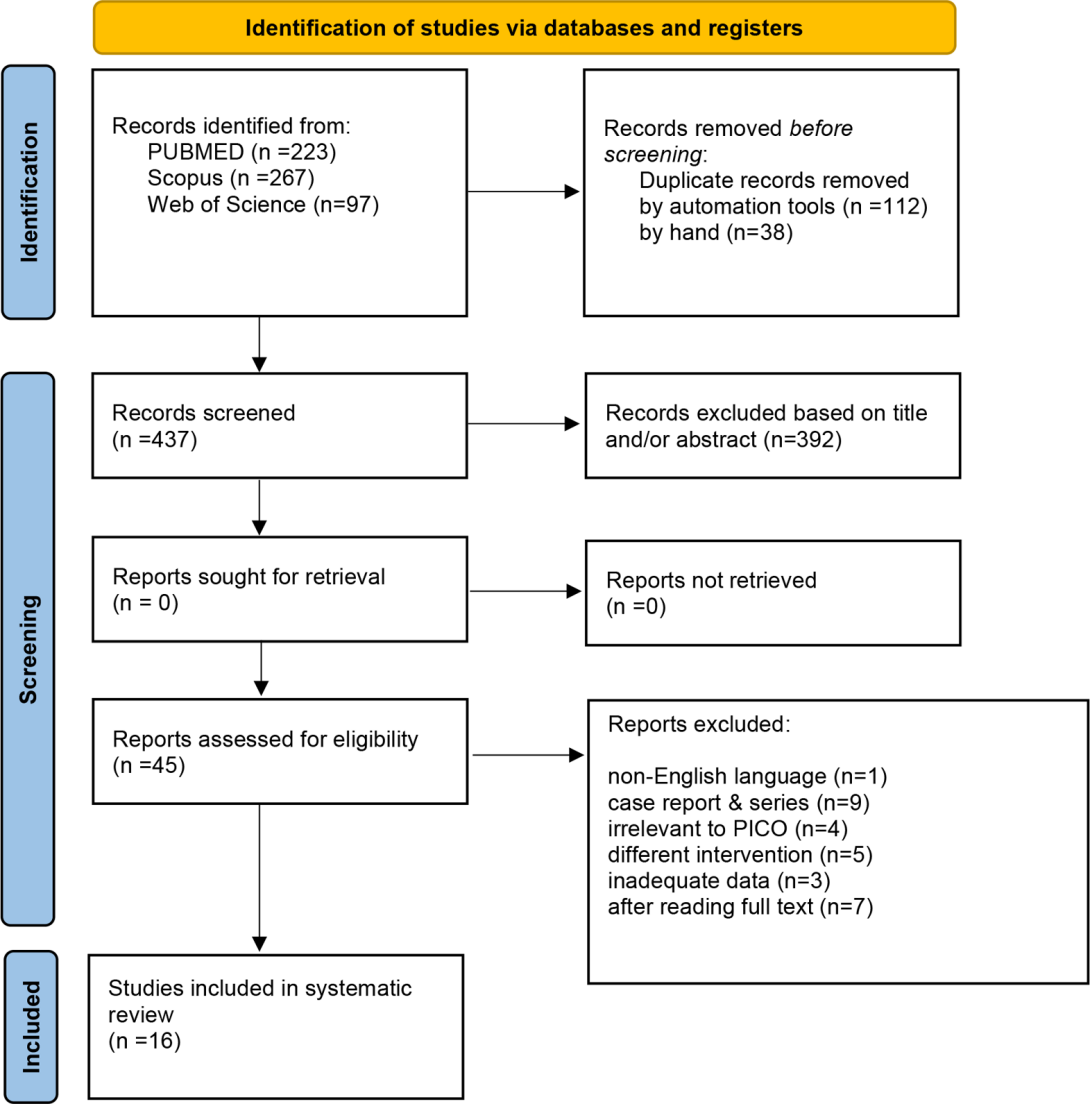
PRISMA Flow Chart

Our search strategy yielded 587 articles. Of these, 150 were duplicate studies, and 437 were screened. Of these studies, 392 studies were excluded based on their titles and abstracts. 45 records were assessed for eligibility in which 29 were excluded as they did not meet the inclusion criteria. Finally, 16 studies met the criteria for inclusion, and were included (Fig. 1).

### Risk of bias quality assesment (RoB)

All of the included articles have shown a low level Bias (Table 3).

### Certainty of the evidence

Observational studies are associated with a high quality of evidence. This indicates a high confidence in the effect estimate (Table 4).

### Egger’s test for small study effects

The results of egger’s test revealed absence of small study effects across the included studies in the meta-analysis (z value = 0.39, standard error = 2.588, *p* = 0.6992) (Table 5).

**Table 5:** Regression based Egger test for small-study effects H0: beta 1 = 0; no small-study effects

Test for small study effects	beta 1	Std. error of beta 1	z	Prob >|z|
Regression based Egger test	1.00	2.588	0.39	0.6992

### Publication bias

**Fig. 2:**
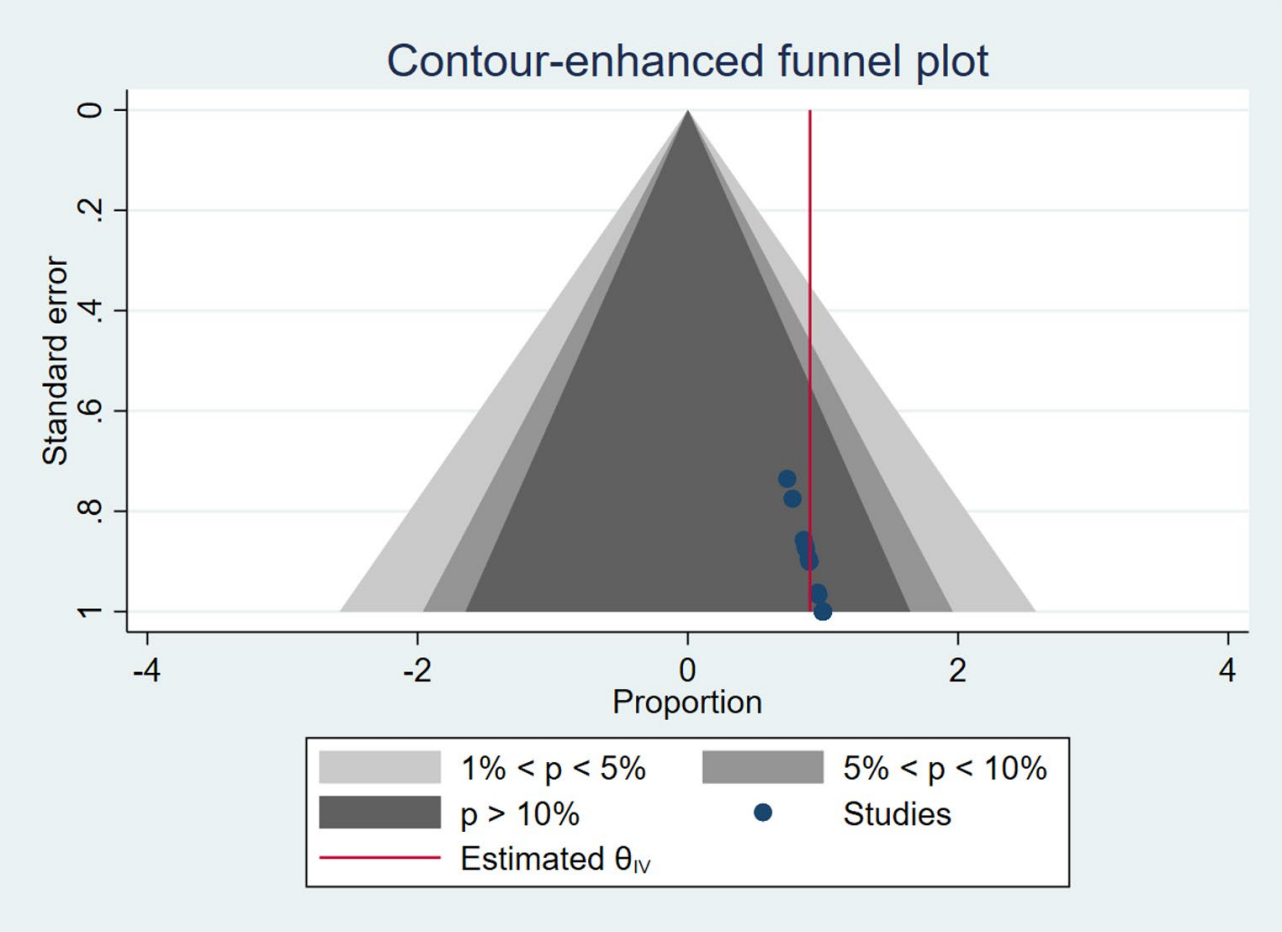
Publication Bias

The contour-enhanced funnel plot suggested that the studies included in this meta-analysis were not strongly influenced by small-study effects or publication bias, as the studies were relatively evenly distributed within the funnel-shaped region. The lack of asymmetry in the plot and the location of the estimated overall effect near the peak of the funnel provide support for the validity of the findings computed from the meta-analysis (Fig. 2).

### Study characteristics

16 studies were included in the current review, Including 13 retrospective [21–33] and 3 prospective [34–36] studies. Totalling 461 patients that underwent tongue flap surgery for the closure of palatal fistula. Size of the fistulas ranged from 1 cm upto 3 cm in diameter with the most common location of the fistula was the anterior palate. Follow up period ranged from 3 weeks to 2 years.

### Succes rate of tongue flap reconstruction of Oro-Antral fistulas

**Fig. 3:**
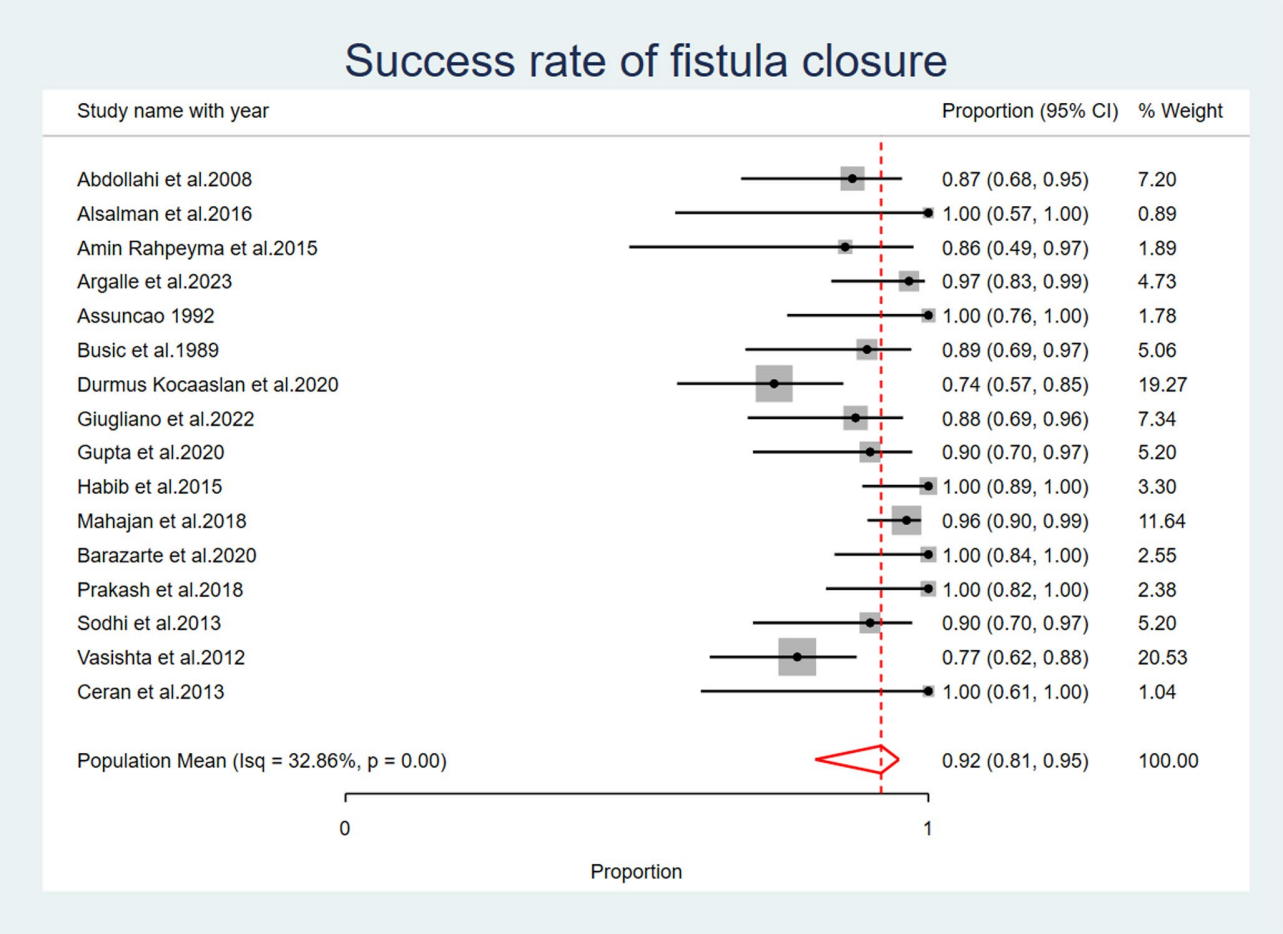
Success Rate of Fistula Closure

Out of 461 flaps performed 359 flaps were successful in the closure of the oro-antral fistula with a total success rate of 77.8% with 102 (22%) flaps failed in the closure of the fistula, with an overall success rate of 0.92 (95% CI: 0.81, 0.95), with significant heterogeneity observed across studies (I2 = 32.86%, *p* < 0.001). A random effects model was used to account for this heterogeneity (Fig. 3) (Table 5).

### Dorsal tongue flaps (DTF) vs. posterior tongue flaps (PTF)

Out of the 16 included studies, only one study (Mhajan et al., 2018) [28] did not report the type of tongue flap used in reconstructing the palatal fistula.

301 patients had dorsal tongue flaps, in which 239 (79.4%) flaps were successful in the closure of the fistula with 62 (20.5%) flaps failed. Out of 301 patients treated with DTF 70 patients (23.2%) experienced complications following the reconstruction of the palatal fistulas using DTF including decrease in hypernasality (16 patients), flap dehiscence (15), total flap detachment (12 patients), partial flap dehiscence (8 patients), total flap dehiscence (7 patients) recurrence of the fistula (7 patients), total flap necrosis (4 patients), complete flap rejection (3 patients), partial flap rejection(3 patients), bleeding from the flap (3 patients), temporary anterior venous congestion (2 patients), partial flap necrosis (2 patients), regurgitation of food/ fluids (2 patients) and sloughing (1 patient) 7 patients had posterior tongue flaps, in which 6 (85.7%) flaps were successful in the closure of the fistula with only 1 flap have failed. Out of 7 patients treated with PTF 6 patients experienced tongue assymetry following the procedure (Table 2). Tongue flap used for fistula closure revealed that the group mean proportion for studies using a dorsal anterior tongue flap was 0.91 (95% CI: 0.80, 0.95). Only one study assessed success rate of palatal fistula closure using a posterior lateral tongue flap which was computed to be 1.00 (95% CI: 0.76, 1.00). There was one study that did not specify the type of tongue flap used, with proportion of success rate computed to be 0.96 (95% CI: 0.90, 0.99).

### Incidence of flap detachment

**Fig. 4:**
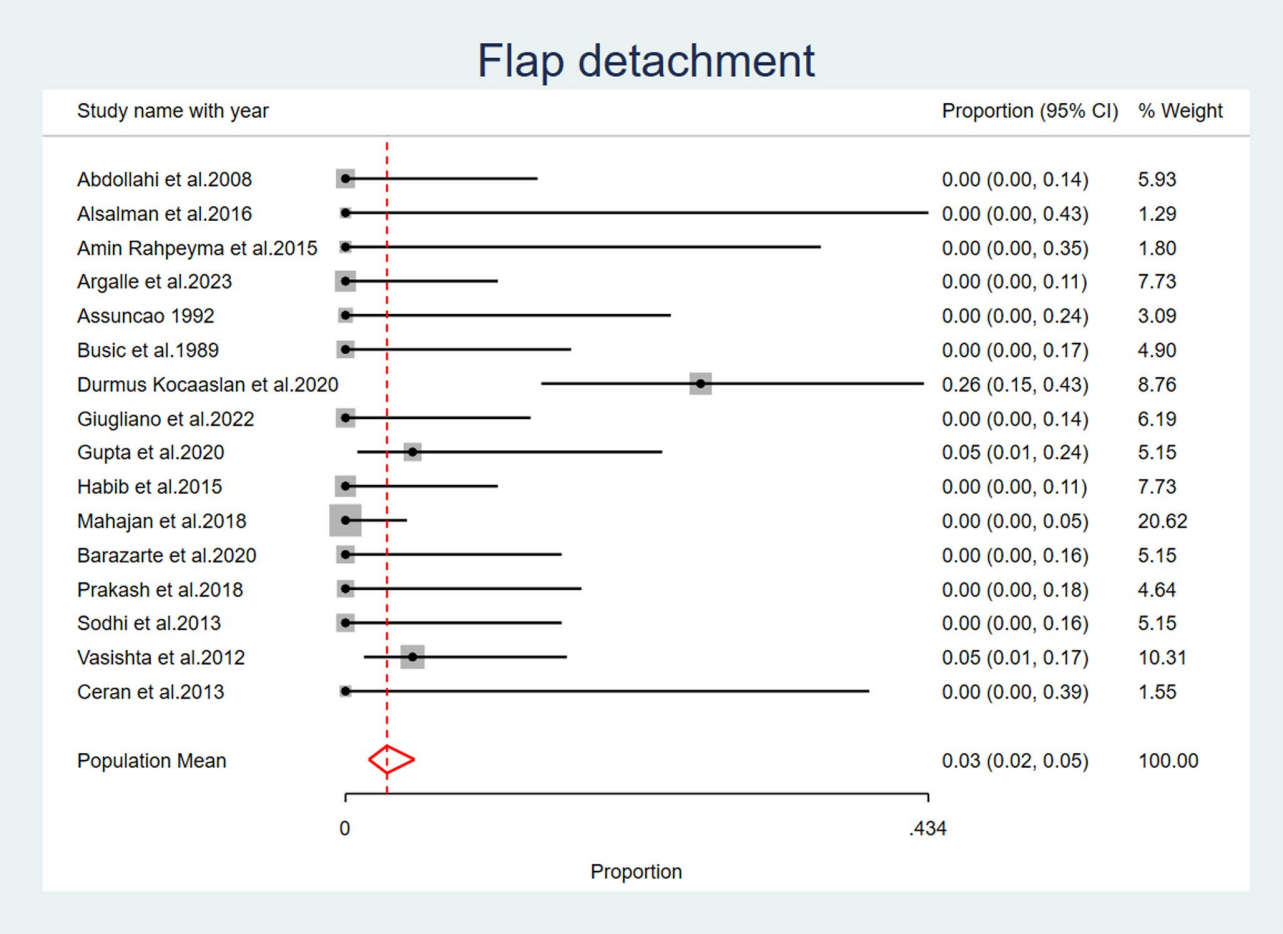
Flap Detachment

Flap detachment was reported as an observed complication after palatal fistula closure only in three of the included studies (Durmus Kocaaslan et al. 2020) [22], (Vasishta et al. 2012) 32] (Gupta et al.2020) [34]. The overall population mean proportion of flap detachment was computed to be 0.03 (95% CI: 0.02, 0.05), as a fixed effects model was used to pool the results due to the low heterogeneity observed across the included studies (I^2^ = 3.14%, *p* = 0.24) (Fig. 4), (Table 6).

**Table 6:** Likelihood ratio test to check fit of random effects and the fixed effects model

Study variable	DF	Chi square statistic	*p* value	Tau^2^	I^2^ tau
Success of fistula closure	1	9.63	< 0.001**	0.73	32.86
Post-operative bleeding	1	1.85	0.09	2.64	6.18
Partial necrosis of flap	1	0.59	0.22	0.90	7.44
Flap dehiscence	1	18.65	< 0.001**	3.19	10.55
Recurrence of fistula	1	1.40	0.12	1.11	8.41
Flap detachment	1	0.82	0.24	2.82	3.14

*p < 0.05 is statistically significant

**p < 0.01 is statistically highly significant

### Incidence of flap dehiscence

**Fig. 5:**
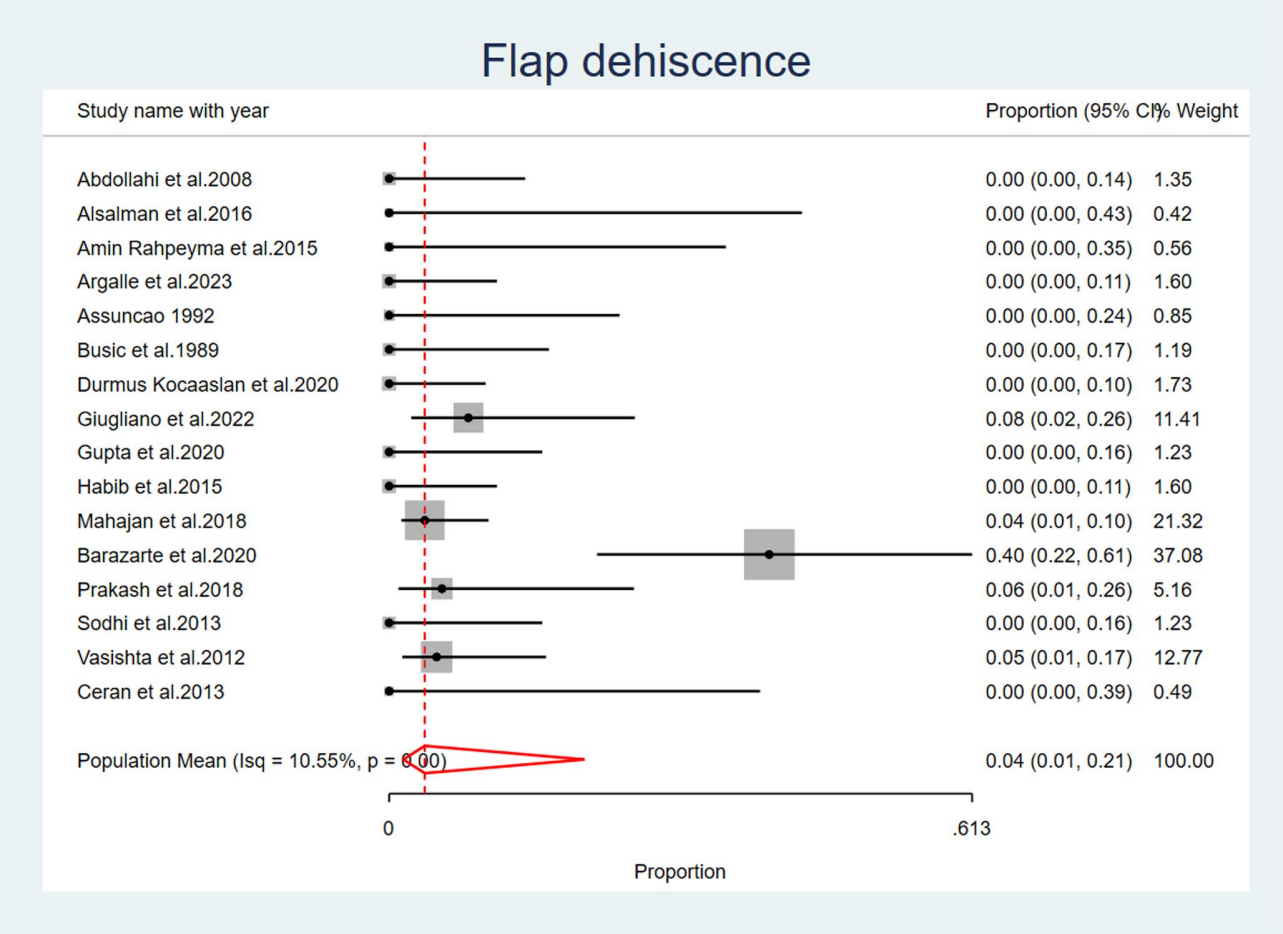
Flap Dehiscence

Flap dehiscence was reported as an observed complication after palatal fistula closure in five studies, (Barazarte et al. 2020) [26], (Mahajan et al. 2018) [28] (Prakash et al. 2018) [29], (Giugliano et al. 2022) [31] (Vasishta et al. 2012) [32]. The overall population mean proportion of flap dehiscence is 0.04 (95% CI: 0.01, 0.21), with significant heterogeneity observed across studies (I2 = 10.55%, *p* < 0.001); a random effects model was used to account for this heterogeneity (Fig. 5) (Table 6).

### Flap necrosis

**Fig. 6:**
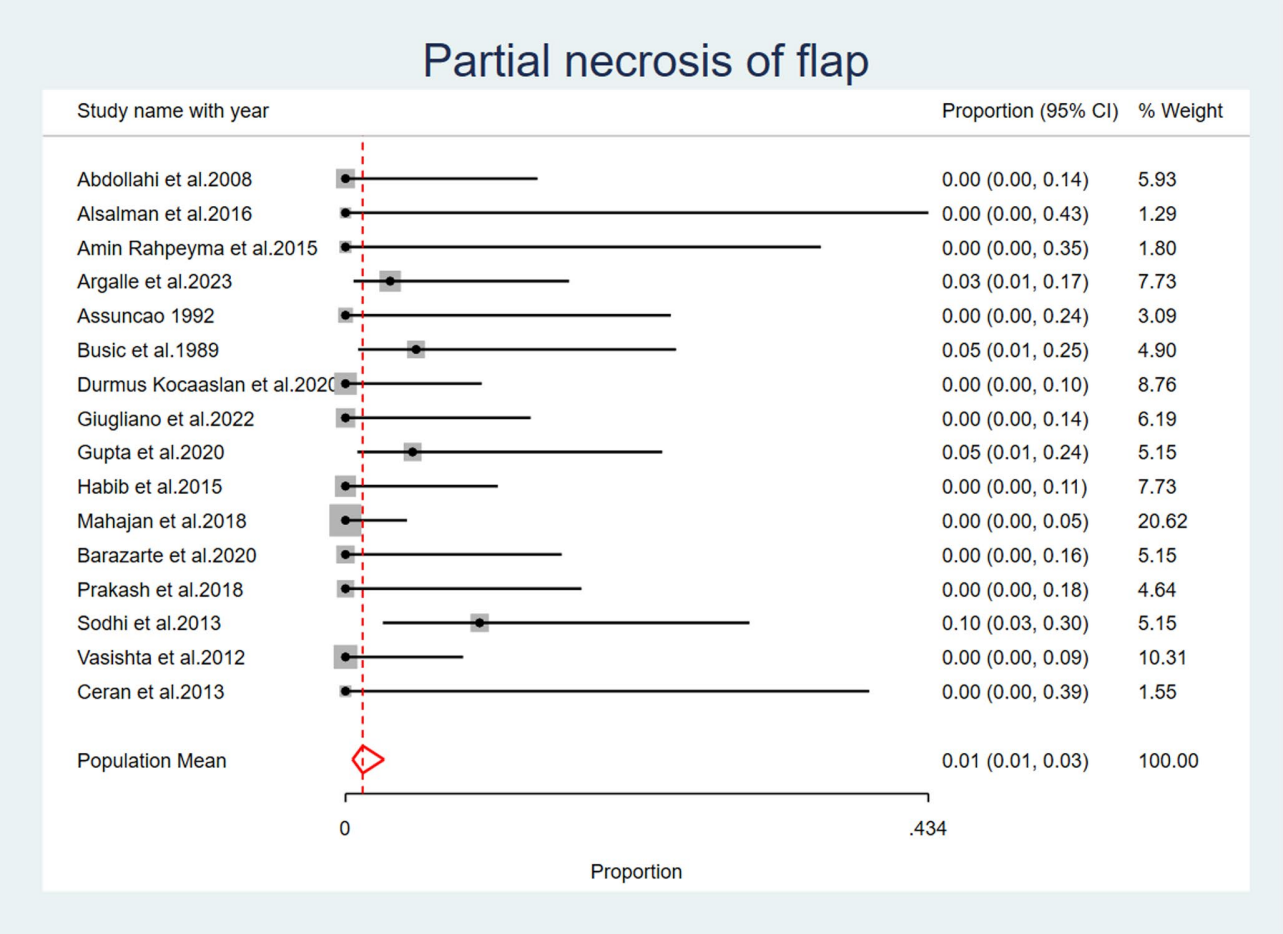
Partial Flap Necrosis

Partial necrosis of the flap was reported as an observed complication after palatal fistula closure in four studies: (Busic et al. 1989) [21] (Argalle et al. 2023) [30], (Gupta et al. 2020) [34], (Sodhi et al. 2013) [36]. The overall pooled proportion of partial necrosis of the flap across all studies is 0.01 (95% CI: 0.01, 0.03) as a fixed effects model was used due to the low heterogeneity observed across the included studies (I2 = 7.44%, *p* = 0.22) (Fig. 6) (Table 6).

### Postoperative bleeding

**Fig. 7:**
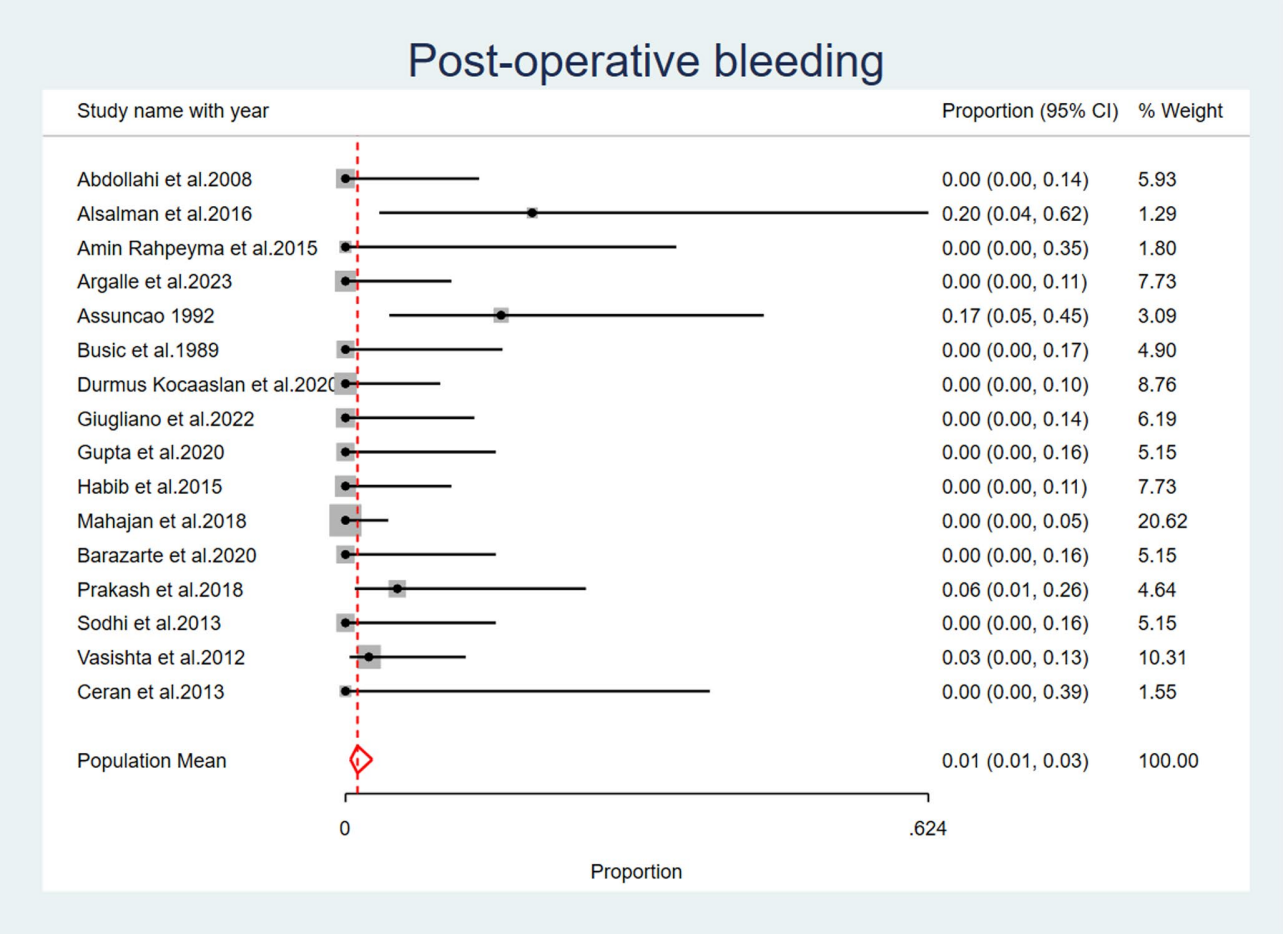
Post Operative Bleeding

Post-operative bleeding was reported as an observed complication after palatal fistula closure in four studies: (Alsalman et al. 2016) [27], (Prakash et al. 2018) [29] (Assuncao 1992) [33], (Vasishta et al. 2012) [32]. The overall pooled proportion of post-operative bleeding across all studies is 0.01 (95% CI: 0.01, 0.03). A fixed effects model was used to calculate this pooled proportion, as the heterogeneity across studies was low (I2 = 6.18%, *p* = 0.09) (Fig. 7) (Table 6).

### Recurrence of fistula

**Fig. 8:**
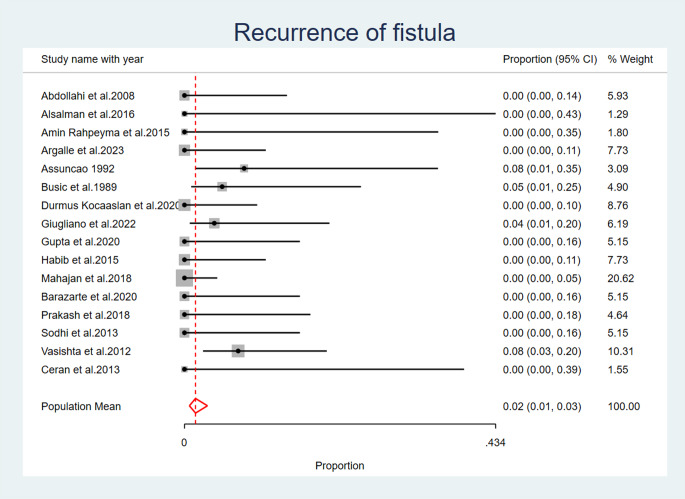
Recurrence of Fistula

Recurrence of fistula was reported as an observed complication after palatal fistula closure in four studies: (Busic et al. 1989) [21], (Giugliano et al. 2022) [31], (Vasishta et al. 2012) [32], (Assuncao 1992) [33]. The overall pooled proportion of fistula recurrence across all studies is 0.02 (95% CI: 0.01, 0.03). A fixed effects model was used to calculate this pooled proportion, as the heterogeneity across studies was low (I2 = 8.41%, *p* = 0.12) (Fig. 8) (Table 6).

### Location of the oro-antral fistula in the outcome of tongue flap reconstruction

**Fig. 9:**
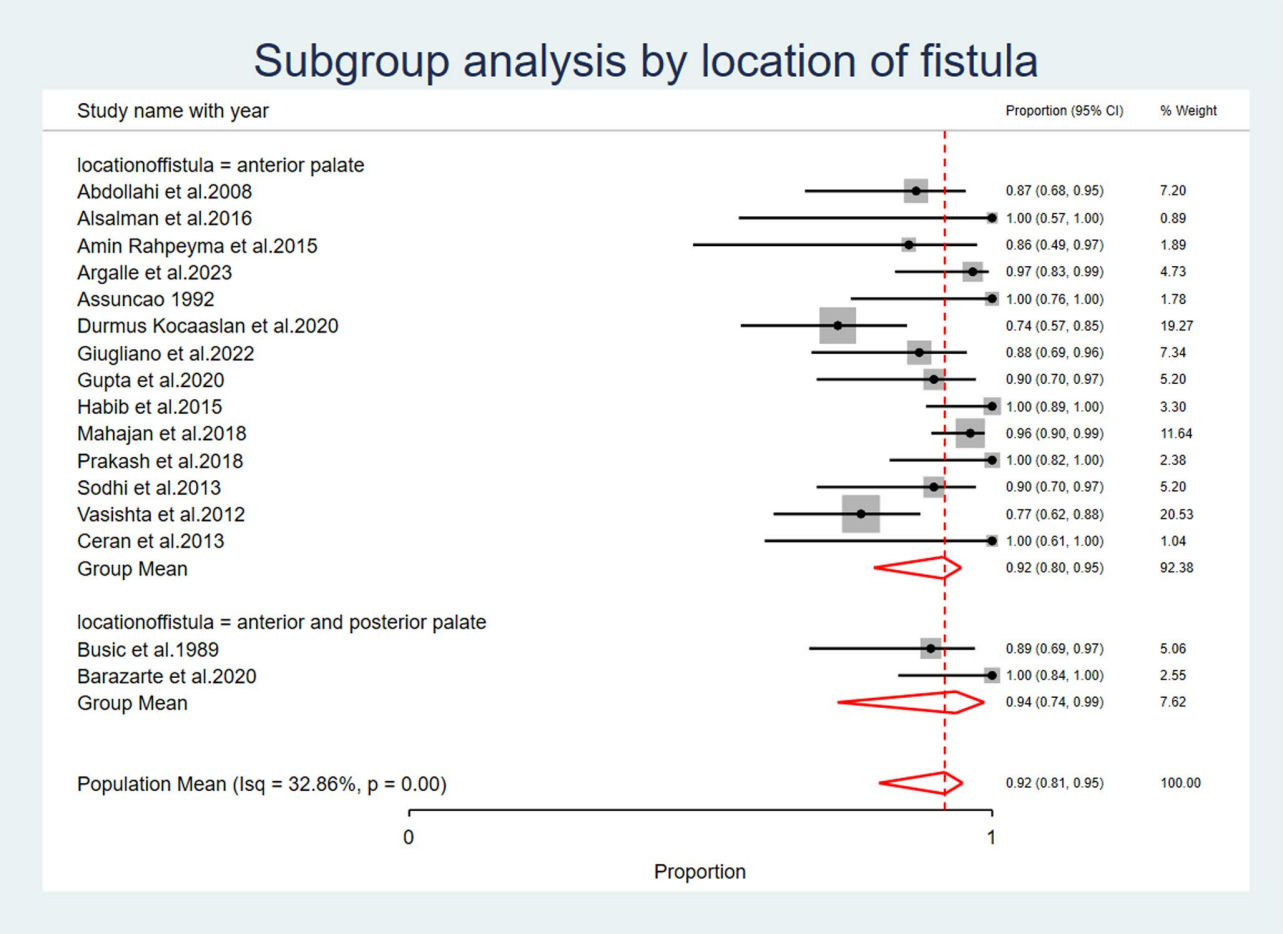
Location of the Oro-Antral Fistula in the outcome of Tongue Flap Reconstruction

Location of fistula revealed that the group mean success rate for closure of fistula located in anterior palate was 0.92 (95% CI: 0.80, 0.95) and for fistulas involving both anterior and posterior palate was 0.94 (95% CI: 0.74, 0.99). The success rate for closure of fistula located in anterior palate (92%) was slightly lower than the success rate for closure of fistula involving both anterior and posterior palate (94%). However, the confidence intervals for the two subgroups overlap, suggesting the difference in success rates was not statistically significant (Fig. 9) (Table 7).

**Table 7:** Meta regression

Predictors affecting overall effect size	Coefficient	Std. error	z	*P*>|z|	95% confidence interval	
					Lower	Upper
1. Type of tongue flap used						
Dorsal anterior tongue flap	(Baseline category)	-	-	-	-	-
Posterior lateral tongue flap	0	Could not be computed due to less number of observations	-	-	-	-
Not specified	0	Could not be computed due to less number of observations	-	-	-	-
2. Study type						
Retrospective clinical study	-0.146	0.063	-2.32	0.020*	-0.269	-0.023
3. Average length of palatal fistula(in cms)	0.151	0.066	2.28	0.022*	0.021	0.281
4.Average width of palatal fistula (in cms)	-0.106	0.057	-1.85	0.065	-0.219	0.007
5. Follow up period (in weeks)	-0.001	0.001	-0.71	0.475	-0.002	0.000
6. Location of fistula						
Anterior and posterior palate	0.273	0.238	1.15	0.251	-0.193	0.740
7. Previous attempts of fistula closure	0.000	0.002	0.13	0.896	-0.004	0.005
8. Constant	0.902	0.086	10.45	0.000**	-0.733	1.071

Method: REML Residual heterogeneity: Tau^2^ = 0.001319 I^2^ (%) = 14.67

H^2^ = 1.52 R-squared (%) = 55.36 Wald chi^2^ (6) = 11.39Prob > chi^2^ = 0.0770

Test of residual homogeneity: Q _res = chi^2^(2) = 2.81 Prob > Q_res = 0.2455

*p < 0.05 is statistically significant

**p < 0.01 is statistically highly significant

### Oro-antral fistula size in the outcome of tongue flap reconstruction

The average length of the palatal fistula was positively associated with the overall success rate (coefficient = 0.151, *p* = 0.022). This suggested that patients with larger fistulas tend to have a better success rate. The average width of the palatal fistula was negatively associated with the overall pooled success rate, although the association was only marginally significant (coefficient = -0.106, *p* = 0.065). This indicated that studies with wider fistulas may tend to report lower success rate. The follow-up period was not significantly associated with the overall success rate (coefficient = -0.001, *p* = 0.475), suggesting that the length of follow-up does not have a significant impact on the reported effects. (Table 7).

### Speech assesment scores, hypernasality and nasal emission outcomes post-operatively

**Fig. 10:**
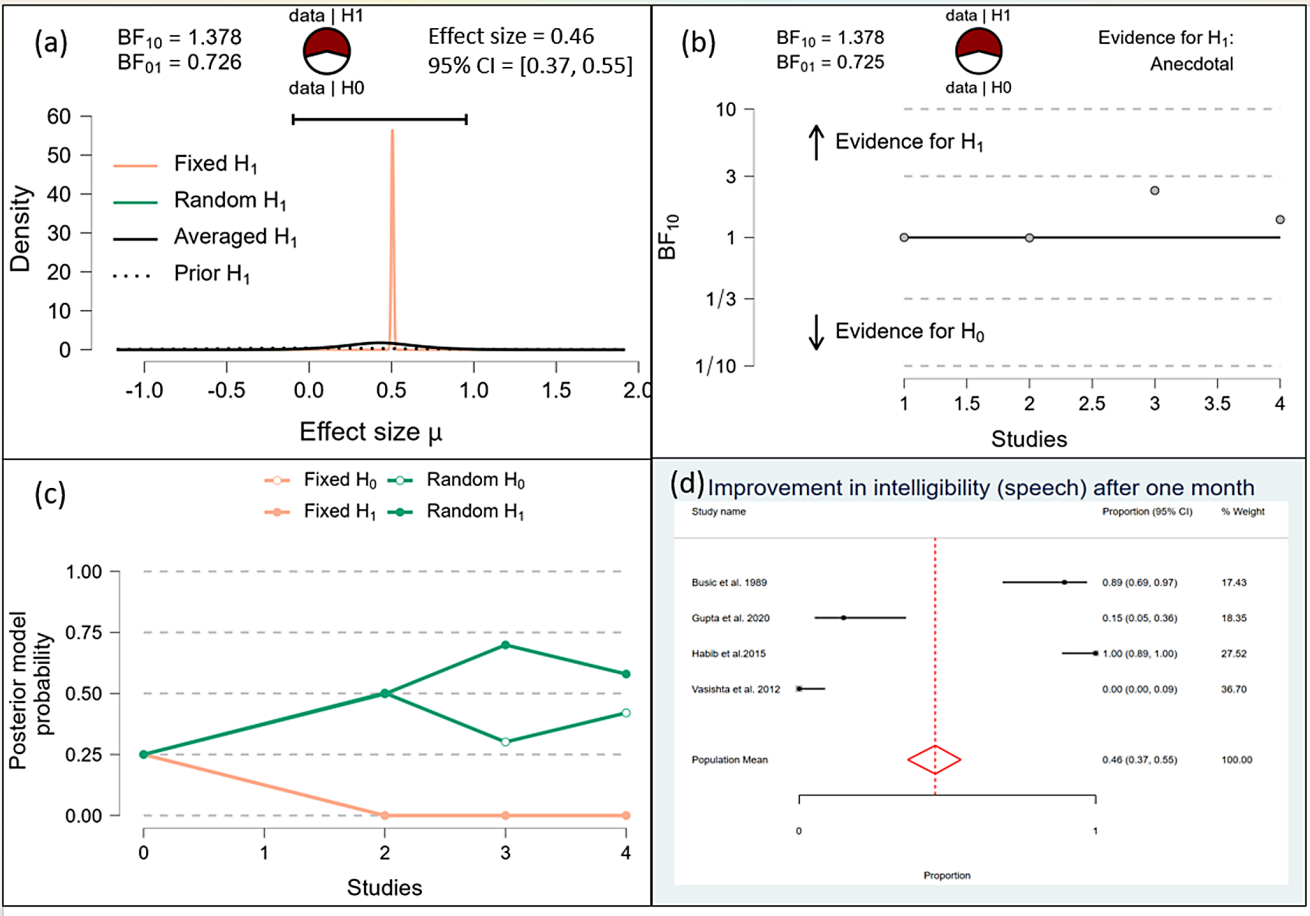
Improvement of Speech after one month

**Fig. 11:**
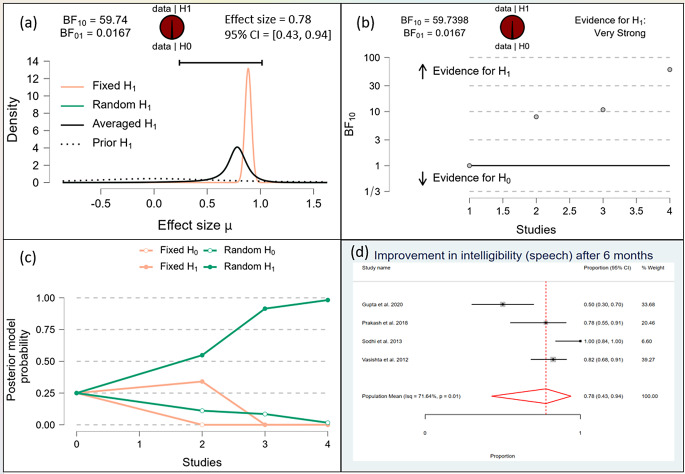
Improvement of Speech after 6 months

**Fig. 12:**
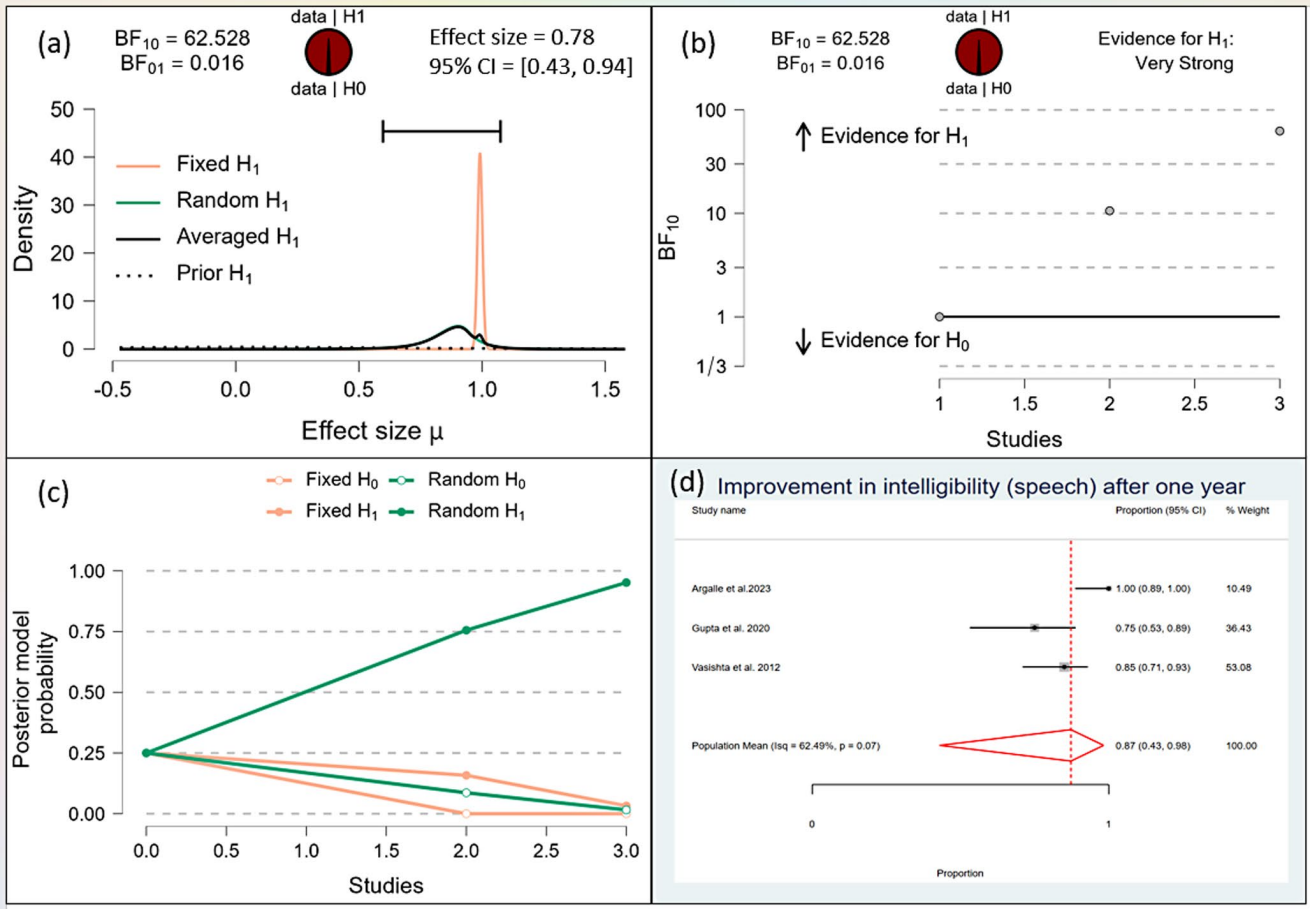
Improvement of Speech after 1 Year

**Fig. 13:**
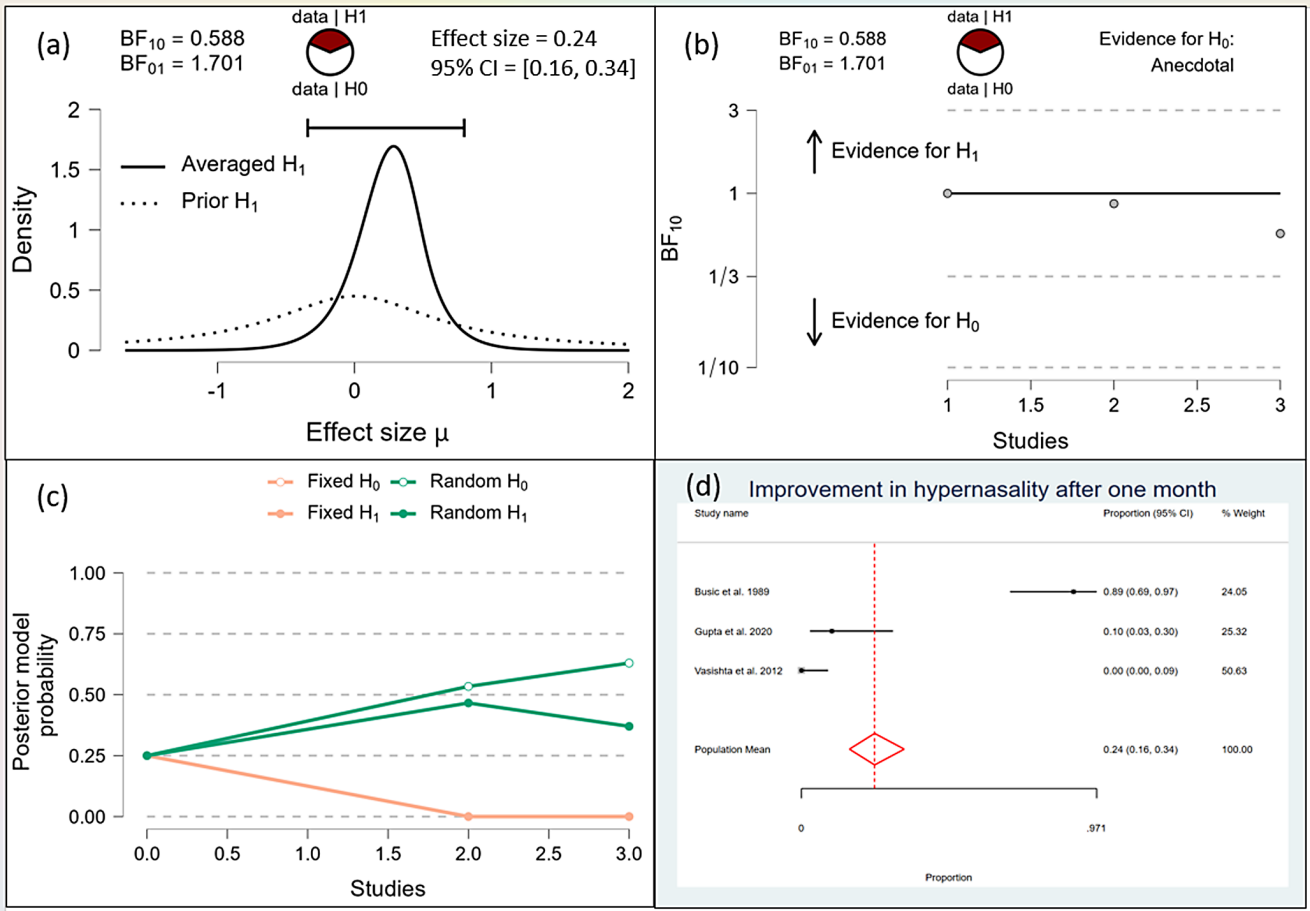
Improvement of Hyper-nasality after one month

**Fig. 14:**
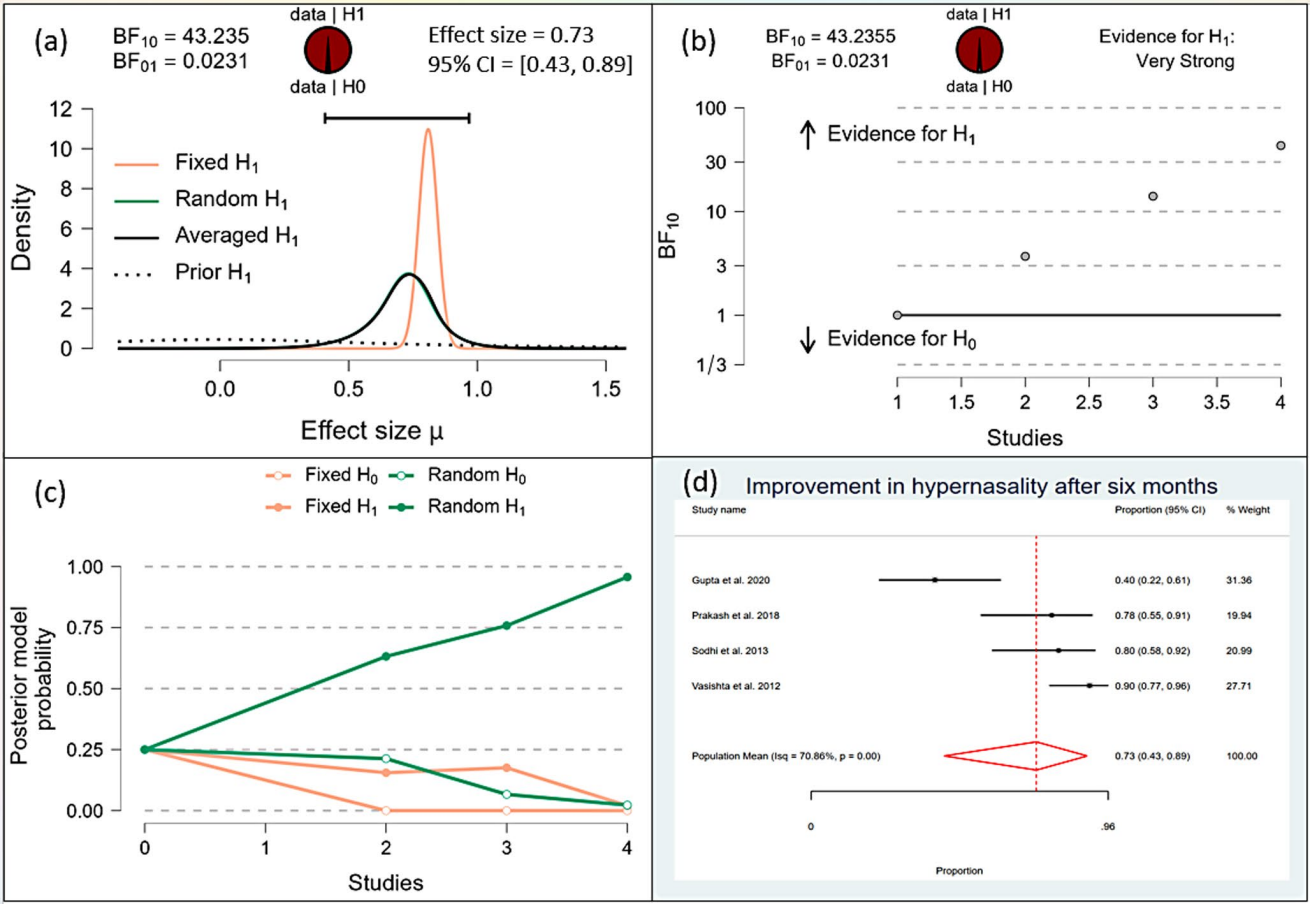
Improvement of Hyper-nasality after 6 months

**Fig. 15:**
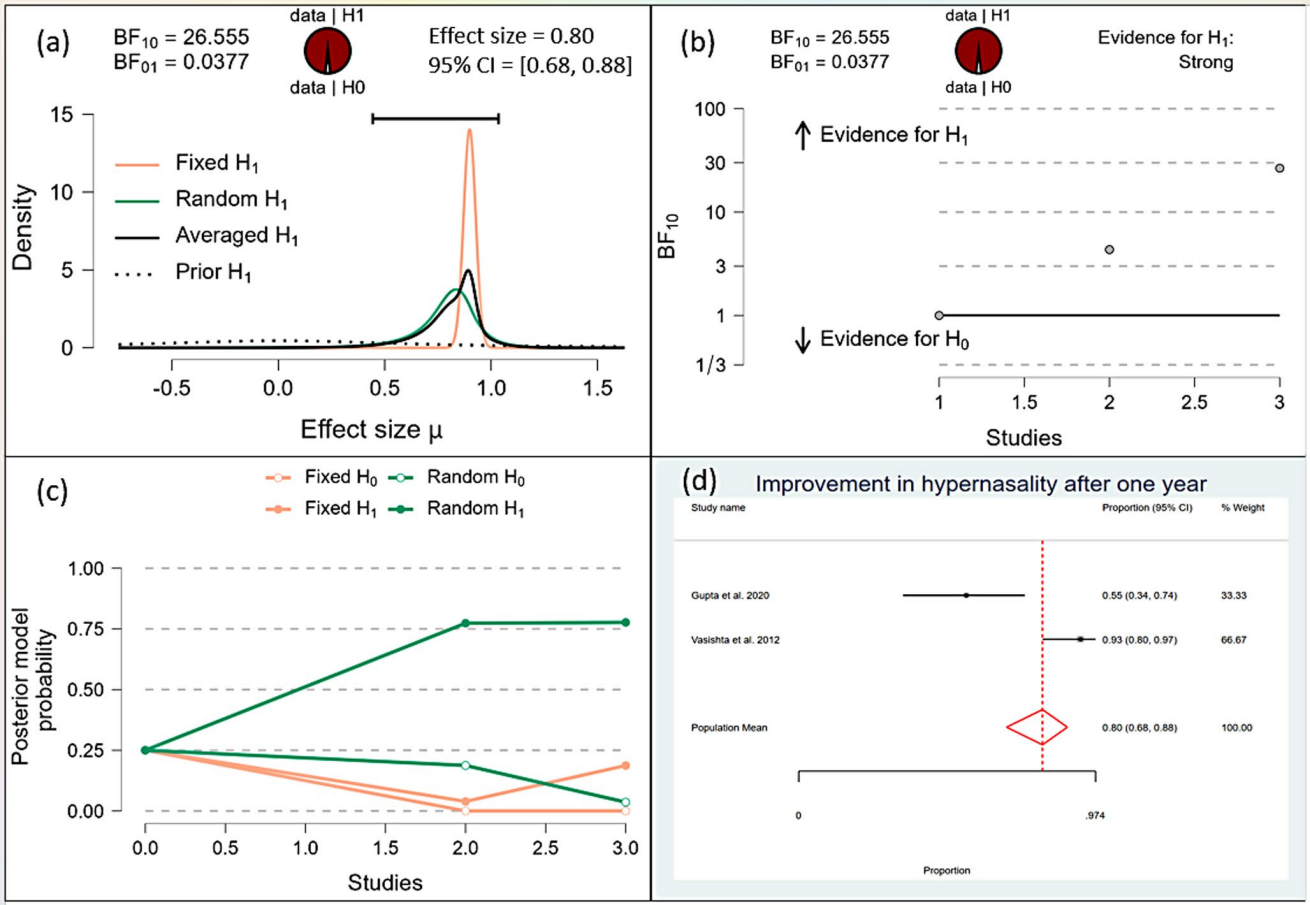
Improvement of Hyper-nasality after 1 year

**Fig. 16:**
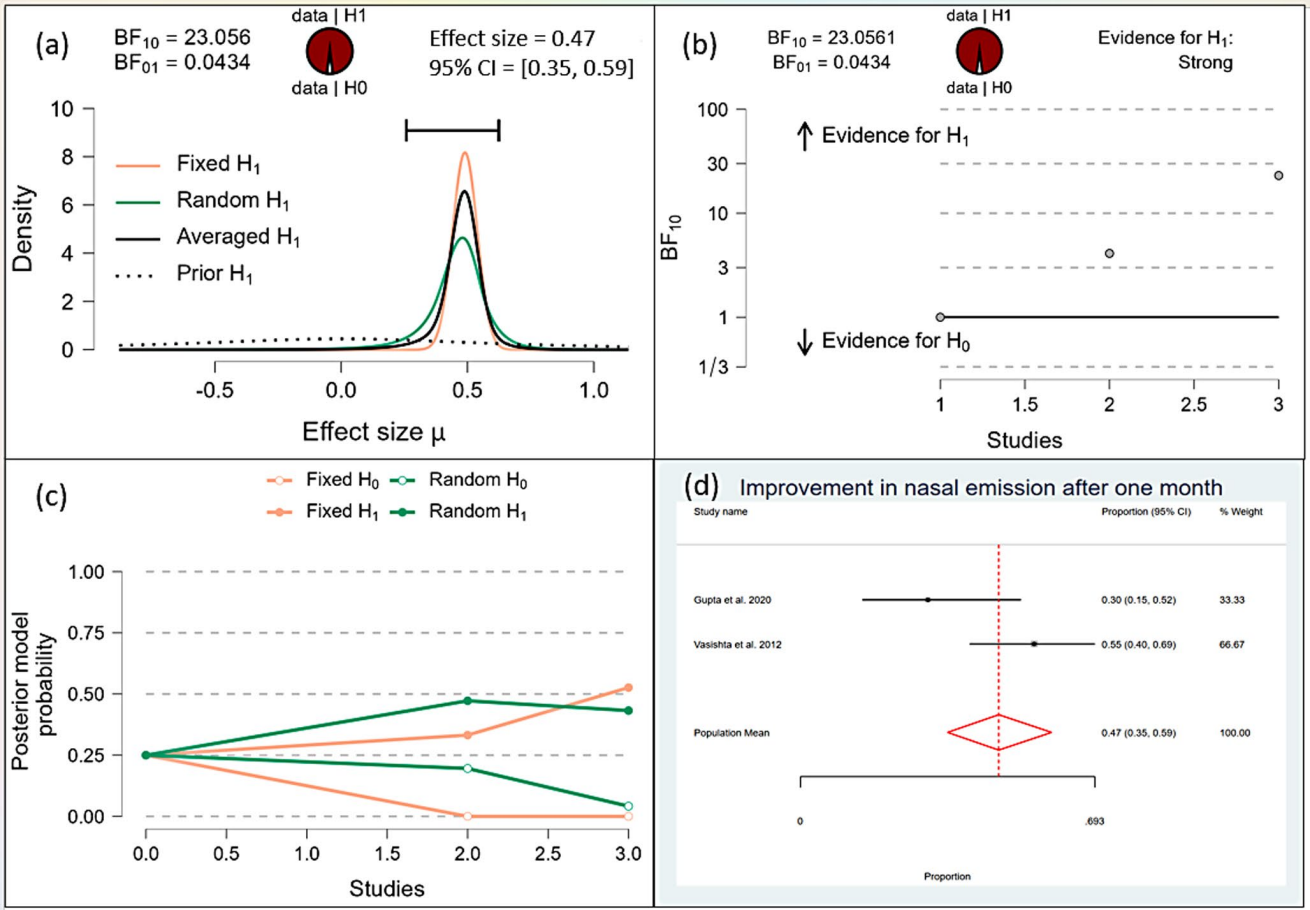
Improvement of Nasal Emission after one month

**Fig. 17:**
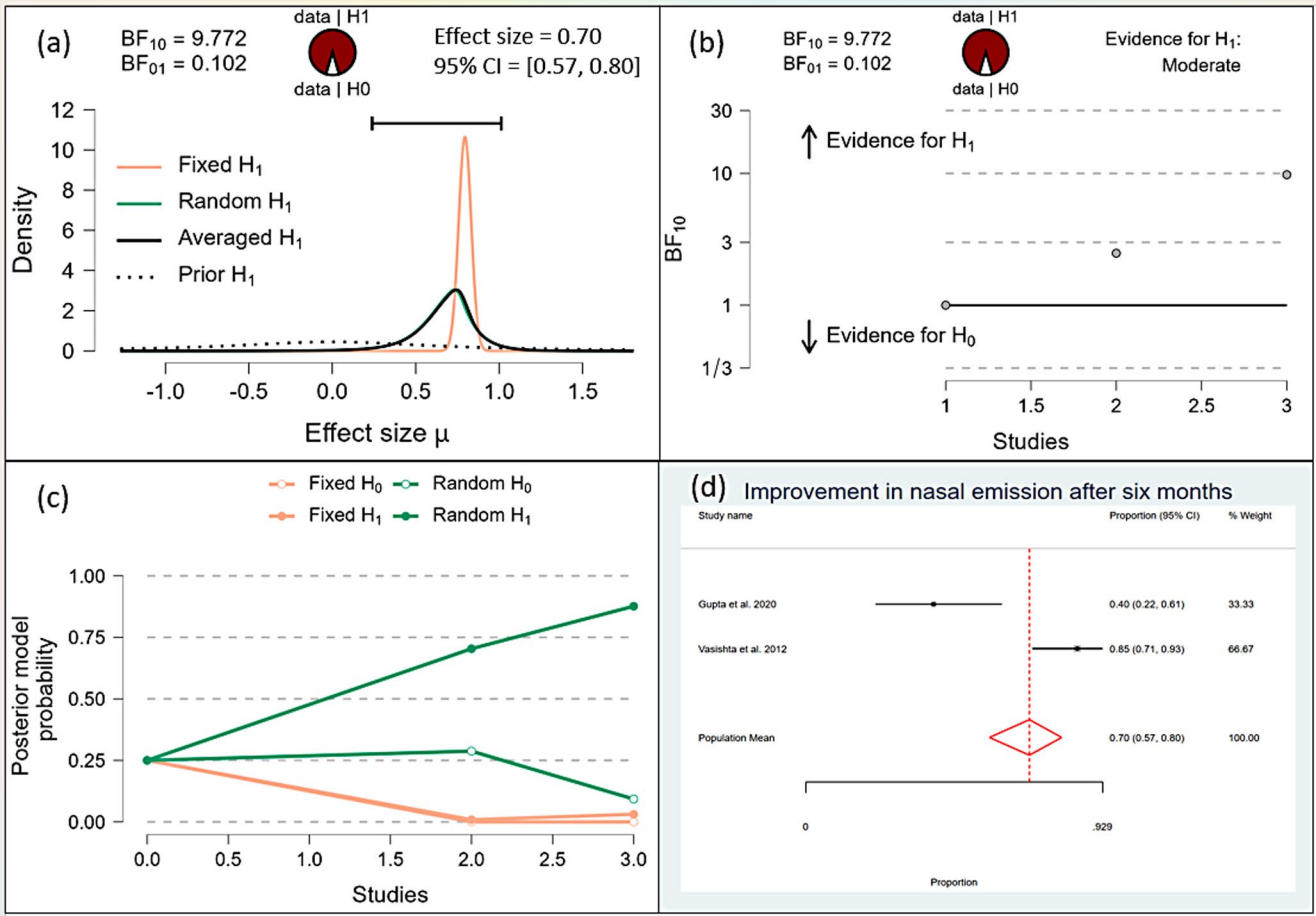
Improvement of Nasal Emission after 6 months

**Fig. 18:**
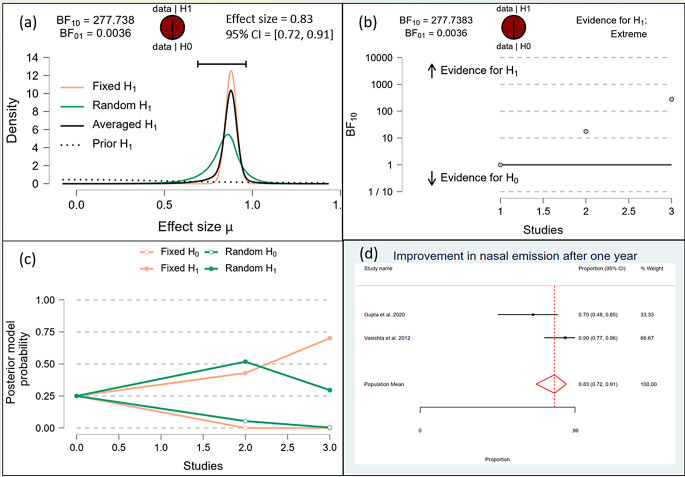
Improvement of Nasal Emission after 1 Year

After one month, the Bayesian analysis provided mixed evidence for the improvement in speech intelligibility, with anecdotal support for the alternative hypothesis (BF10 = 1.378) but wide confidence intervals indicating high uncertainty. There was anecdotal evidence for no effect on hypernasality (BF10 = 0.588), and moderate evidence for improvement in nasal emission (BF10 = 9.708). After six months, the analysis showed strong evidence for improvement in speech intelligibility (BF10 = 59.7398) and hypernasality (BF10 = 35.619), along with strong evidence for improvement in nasal emission (BF10 = 23.056). By one year, the evidence became very strong for the improvement in speech intelligibility (BF10 = 62.528) and hypernasality (BF10 = 62.528), and there was extreme evidence for the improvement in nasal emission (BF10 = 277.738). (Figs. 10, 11, 12, 13, 14, 15, 16, 17, 18).

## Discussion

The success rate for closure of fistula located in anterior palate (92%) was slightly lower than the success rate for closure of fistula involving both anterior and posterior palate (94%). However, the confidence intervals for the two subgroups overlap, suggesting the difference in success rates was not statistically significant. These successful results are magnificent results if compared to local flaps that is impossible to be used in closure of large or even small palatal defect due to teared, scared, and difiecient tissues amount in cases of palatal defects [2, 5].

Both dorsal and posterior tongue flaps provide the main target as soft tissue graft in reconstruction and closure of palatal defects based on being of the same mucosa of the palatal defect in addition to the high vascularity of tongue mucosa either in the dorsal or positerior tongue flap. Bracka (1981) [37] confirmed the importance of the width of the dorsal graft to be half to two-thirds of dorsal tongue width in order to ensure a good blood supply with its submucosal plexus in posterior based type that enriched the flap and improve the healing. However, for anterior based type, the plexus of the Ranine Arch at the tongue tip in addition to dorsal arteries permits perfect healing [37], while regarding the posterior tongue flap, 1/3 of the tongue width could be included based on the the width of the defect. Moreover, it could be incised from below in longitudinal direction to convert the thick, narrow posterior flap to a thin wide one with its axial blood supply [26].

Guzal and Altintas also recommend the thickness of tongue flap that includes the mucosa and approximately 5 millimetres of the underlying muscle to ensure inclusion of the rich submucous plexus [38]. The length of dorsal tongue flap of about 5–6 cm with the base of the flap 2.5 to 3 cm and about 1 cm far from circumvalate papille allow tongue movement and enable healing without IMF, only bite blocks were used to open the airway and to prevent kinking of the tongue flap [39, 40]. In addition, the length of posterior tongue flap has a successful role by its length with simplicity that allows easily elevation of flap from lateral side of tongue that does not limit the mobility of remaining tongue, with a good choice for reconstruction of central palatal defects without need for of maxilla-mandibular fixation [24].

In the present analysis, five studies reported flap dehiscence complication following palatal fistula repair. Three studies reported flap detachment. Partial necrosis of the flap was noted in four investigations. Four studies reported post-operative bleeding. Four studies have documented the observation of fistula recurrence. This complication can be reduced by proper selection of the type of the tongue flap posterior or dorsal with anterior or posteriorly pedicled to allow proper healing without tension.

As a mechanical role the flap base must lies beneath the posterior border of the fistula with the tongue in the neutral position and with enough length that fill the antero-posterior dimension of the fistula with at least 1 cm spare for proper coverage with freedom movement of tongue [38, 41]. Moreover, the size of dorsal flap can be modified to decrease tension and detachment by making it fork or Y shape form to cover palatal and alveolar clefts simultaneously. Kim proved the role of fork and Yshape flap in closure of large palatal defects on 14 patients with proper healing without detachment [39].

Durmus Kocaaslan et al. 2020 recorded another cause of detachment of dorsal tongue flap through 15 years retrospective study on tongue flaps surgeries on 34 patients of both sex with a mean age of 11.7 ± 6.9.The analysis of data showed that the detachment of the tongue flap was the main post operative complication that appear in seven male patients aged 6 years old with repeated failure after resuturing, while for posterior tongue flap, the detachment in early postoperative period represents a lesser complication as bone suture technique can be used for suspending the posterior tongue flap to the palate preventing the detachment [22].

The main disadvantage of posteriorly based lateral tongue flap for closure of palatal fistula is the thick nature of the flap, making it necessary to carry out a third surgery in some cases. Additionally, asymmetrical tongue shape is associated with speech problem that does not improve with time [24], while for dorsal tongue flap there is no interference with speech, with no defect in the tongue symetry. Also, oral hygiene and mastication remain unimpaired, with no sensory or gustatory disability following this procedure [40]. The main disadvantages of both dorsal and posterior tongue flap are that of the requirement of a 3-week interval between tongue flap elevation and division, as well as the fact that bone graft must be postponed until after approximately 8 months in case of cleft alveolus repair [39].

Inspite of the type of the tongue flap used in closure of palatal defect, the average length of the palatal fistula was positively associated with the overall success rate (coefficient = 0.151, *p* = 0.022). This suggested that patients with larger fistulas tend to have a better success rate. The average width of the palatal fistula was negatively associated with the overall pooled success rate, although the association was only marginally significant (coefficient = -0.106, *p* = 0.065). This indicated that studies with wider fistulas may tend to report lower success rate and this result is in accordance with the concept that larg defect allow proper watertight seal of the tongue flap to the defect [40]. In a prospective study of 20 patients with anterior palatal fistulae (larger than 5 mm), Sodhi et al. reported that successful closure was achieved in 90% of cases. The anteriorly based dorsal tongue flap is a well-accepted method for treating cleft palate patients with oronasal fistulae [36].

Moreover, Zhao-hui Yang et al. 2019 [42] used tongue flaps in 5 patients to close large palatal fistulas, with all flaps surviving without complications, and the postoperative appearances considered to be either excellent or satisfactory with no recurrent fistulae in all patients.

In 65% of the included studies, dorsal tongue flap was employed more frequently than the reported posterior lateral tongue flap. Tongue flap is successful in closure large palatal defect with enough length, width, even in a very thin form of flap that reach to 3–5 mm thick along its length with the thicker part at its base to maintain blood supply taking in account the proper position of flap in relation to defect as an important mechanical factor. It is recommended that using of any form of diathermy or injection of adrenalin into the flap is prohibitted both at the donor or recipient sites.

Detachment, and necrosis and fistulous opening are more common complications with dorsal flap while for posterior tongue flap tongue assymetry and speech defect are the main problems that accompany the posterior flap. Age of patients is recommended to be higher than 7 years old especially in dorsal tongue flap to overcome any detachment complication. Limitations of this study include limited amount of randomized controlled trials, incomplete data, missing patient information.

## Conclusion

This study highlights that tongue flaps, particularly dorsal tongue flaps (DTF) and posterior tongue flaps (PTF), are effective options for palatal fistula closure, demonstrating high success rates and a favorable complication profile. Nonetheless, additional research is necessary to explore the potential of these techniques for closing oroantral fistulas (OAF). Further investigations should employ randomized controlled trials with larger patient cohorts and extended follow-up periods to comprehensively evaluate the efficacy, complication rates, and long-term outcomes of DTF and PTF in OAF treatment.

## Data Availability

No datasets were generated or analysed during the current study.
